# Does Family Migration Affect Access to Public Health Insurance? Medical Insurance Participation in the Context of Chinese Family Migration Flows

**DOI:** 10.3389/fpubh.2021.724185

**Published:** 2021-09-30

**Authors:** Luchan Liu

**Affiliations:** School of Sociology and Population Sciences, Nanjing University of Posts and Telecommunications, Nanjing, China

**Keywords:** public health insurance, family migrants, social welfare, floating population, public health policy

## Abstract

Using 2017 Migrant Dynamic Survey (CMDS) data, logistic regression models were developed to explore the family migration rate on health care participation of floating population. The analysis reveals that 68.69% of the floating population in China moves with at least one family member, but the local health insurance participation rate of them are relative low. However, family migration rate has a significant positive correlation with the health insurance participation of the floating population at the destination, which explains by family support and social integration mechanisms. The higher the degree of family migration, the higher the likelihood of participating in local health insurance system. Age, labor contract types, migration range and cities numbers, health records, and the accessibility of health resources have a significant negative correlation with health care participation of the floating population at the destination; gender, health, marriage, education, hukou types, monthly income, migration history, and move duration have a significant positive correlation. The effect of family migration rate on health care participation is weaker in group in which people are low-educated and signs non-fixed-term contract or gets bottom 50% monthly income or under the no-kids family structure. Potential policies informed by these findings are also explored.

## Introduction

The health and social welfare of migrants has always been a central issue in migration research. Since the 1980s, a large number of rural residents in China have poured into cities to participate in economic and social development actively. The large floating population faces great risks from its mobility while driving China's modernization. Affected by subjective factors such as insufficient health awareness and objective factors such as high health costs, relatively poor living and working environment, the migrant population often faces health problems, including infectious diseases, reproductive system diseases, occupational hazards, and mental health issues (1). Therefore, the overall health of the migrant population in China is poor, and there is a need for improved basic health services and medical security.

Under China's current urban-rural dual structure, the medical security of the floating population is in a precarious state. Although there are institutional arrangements, they are difficult to implement, and migrants cannot enjoy the same social welfare and social rights as urban residents. From 2011 to 2012, data from the China Migrants Dynamic Survey showed that the migrant population's overall medical insurance participation rate was about 69%, while the rate of urban medical insurance participation was about 26%, producing an obvious Matthew Effect (2). From 2013 to 2017, the number of migrant workers participating in social medical insurance was only 82.88%, most of whom participated in their original place of household registration. They mainly participated in the new rural cooperative medical insurance, and there was repeated participation (3). In recent years, it has been found that the medical insurance participation rate of the floating population has significantly increased and the phenomenon of repeated insurance has improved, but 10.9% of the floating population still lack any medical insurance (4).

In response, the 19th National Congress of the Communist Party of China plans to establish a multi-tiered social security system covering all the people. Public medical insurance, an important part of this system, is an essential means of preventing uncertainty and avoiding health risks among the floating population. Improving their participation in extensive and appropriate medical insurance and improving the fairness of basic medical care services will be one of the focal points and challenges in the development and construction of China's social security system in the future.

After a long period of rapid growth, the scale of China's migrant population has begun to enter a period of adjustment. Migration flow has shifted from the initial individual migration to family migration. Although the scientific measurement of family migration remains to explored, the increasing proportion of female, married, or family migrants among the floating population directly reflects this trend (5). The floating population's desire to settle with their spouses, children, or parents and work in cities is bound to bring greater demand for public services and social security at the destination. Under this conflict of demand and supply, it is urgent to explore whether family migration will affect the participation of the floating population in public medical insurance at the destination.

## Literature Review

### Public Health Insurance for Migrants

Many scholars have discussed the participation of migrant populations in medical insurance programs. Studies show that the factors affecting this participation are complicated, including three main aspects: personal, socioeconomic, and migration characteristics.

Most previous studies have stated that age is an important factor affecting the participation of migrants in medical insurance. The older the migrants or the worse their physical condition, the higher the probability they participate in medical insurance (6). However, some studies have shown that age has no significant effect (7). Relevant personal characteristics include gender, marriage, and health status (8–10). In terms of socioeconomic characteristics, having an employment contract has a greater impact than *hukou* (household registration) status in determining whether Beijing's floating population accesses social insurance (11). Having a stable contract and high job stability are also important factors (12). Regarding migration characteristics, the reasons for migration, frequency of returning home, willingness to settle long-term, and destination area characteristics are the main factors affecting the medical insurance participation rate (13, 14).

### Family Migration in China

Family migration is the main trend in China's current and future population mobility. Family migration could explains in two senses: first, the process of family members from the same nuclear family moving from the countryside to the city; second, the rising proportion of the total floating population moving with family members (15). The main migration mode at present is that the husband and wife move with their children or unmarried grown-up children move with their parents; frequently, three generations of family members stay together at the destination (16). In detail, migration patterns could be distinguished for four types: all family members migrate together, husband and wife migrate together, husband or wife and children migrate together, and individuals migrate alone (17). For most migrants, “laddering migration” is the most common form, that is, family members gradually reunite at the destination, finally forming a complete family (18).

The impact of family migration mainly affects employment income, social integration, children's education, and other aspects. Family migration has an impact on the employment of the floating population. The impact of female floating population is more significant for the lower employment rate and more unstable employment (19, 20). Family migration also promotes migrant workers' participation in community activities, which can enhance their psychological and economic integration, but may reduce their willingness to reside in urban areas (21). However, some scholars have drawn the opposite conclusion, arguing that compared with individual migration, some family members who move together or move with their whole family are more inclined to stay for a long time or even settle permanently at the destination (22). The education problem of migrant children are strengthen by the increasing family migration trend. Children either live with their parents in the city or stay behind in their hometown and trapped in a dilemma of separation (23). In addition, some scholars have noticed a connection between family-centered migration and the family welfare of the floating population, but only at the level of policy analysis, it is suggested that the overall welfare of migrant families should be paid attention to when making policy, but there is a lack of empirical evidence (24, 25).

### Current Study

Although the medical insurance participation of the floating population and family migration have received increasing academic attention, there are still relatively few empirical studies on the relationship between these two factors. Current research on this topic still has the following deficiencies. First, when discussing the factors affecting the floating population's participation in medical insurance, the trend of family migration have not been analyzed, even though it has become a consensus among scholars that China's current population mobility shows a family migration trend. Second, existing studies pay more attention to the overall health insurance coverage of China's floating population and less attention to health insurance coverage at the destination. Third, in terms of the measurement of family migration, scholars have not yet unified its definition and there are different measurement standards. Some measurements have been overly simple, and have been unable to reflect family-oriented migration flow fully.

Therefore, this paper will measure both the existence and degree of family migration. It also discusses the impact of family migration on medical insurance participation at the destination, and attempts to provide relevant explanations, in order to provide empirical evidence for the improvement of relevant policies on family mobility and family welfare, and improve the basic well-being and anti-risk capability of migrant families.

## Method

### Data

The data used in this study come from the 2017 Migrant Dynamic Survey (CMDS). These data are from a national sample survey of the floating population, run by the National Health Commission of China. The survey were conducted annually since 2009, covering 31 provinces (regions, municipalities) and Xinjiang Production and Construction Corps, enabling it to fully reflect the status of China's floating population. The survey questionnaire includes data variables relevant to this study, including basic information about the floating population and their family members; mobility range and trend; employment and social security; income, expenditure and residence; basic public health services; and other specific information. Jiangsu province, which contains a large number of immigrants, is a typical area to observe the family migration phenomenon. In 2017, the sample size of Chinese floating population was 169,989. After screening and converting relevant variables and removing missing values, 71,979 valid samples were analyzed.

### Variables

#### Dependent Variable

Table 1 show the definitions and assignment of variables. We investigated whether the floating population participates in medical insurance at the destination. The relevant survey question is “Which of the following social medical insurance programs are you currently enrolled in?,” with four possible types: New Cooperative Medical Scheme, Rural & Non-Working Urban Residents' Basic Medical Insurance, Non-Working Urban Residents' Basic Medical Insurance, and Urban Employees' Basic Medical Insurance. Those who participated in at least one type of medical insurance in their local area (where they currently lived) were defined as those who participated in medical insurance at the destination, while those who answered in the “household registration area” or “other places” were defined as those who did not participate in medical insurance at the destination.

**Table 1:** Definitions and assignment of variables (*N* = 71,979).

	**Variable**	**Definitions of variable**	**Assignment of variables**
Dependent variable	Health care	Whether to participate in medical insurance at destination in NCMS/R&NURMNI/NURBMI/UEBMI	Participated = 1 Not participated = 0
Independent variable	Family migration rate	The proportion of local member number of the total family number	Local member number/total family number
Control variables (individual characteristics)	Age	Age	2017 minus the year of birth
	Gender	Gender	Female = 0 Male = 1
	Health	Subjective health	Healthy = 1 Basically healthy = 2 Unhealthy = 3
	Marriage	The status of marriage	Single = 1 First married = 2 Remarried = 3 Divorce = 4 Widowed = 5 Cohabit = 6
	Education	Education	Primary school or below = 1 Junior high school = 2 Senior high school = 3 Junior college = 4 Bachelor degree or above = 5
	Hukou types	The type of Hukou	Agriculture = 1 Non-agriculture = 2
	Labor contract types	The type of labor contract	Non-fixed-term contract = 1 Fixed-term contract = 2 Not sign contract = 3 Not Applicable = 4
	Monthly income	The monthly salary	Take the logarithm of salary
Control variables (migration characteristics)	Migration history	Years calculated from the earliest migration year	2017 minus the first year of migration
	Move range	The range of migration	Across the province = 1 Across the city in the province = 2 Across the county in the city = 3
	Move duration	The length of time since the latest move (months)	(2017 minus the year of entry current destination)^*^ 12
	Move cities	The number of cities he/she moved	Total number of cities
Control variables (characteristics of current health resources)	Health records	Whether to establish a health record at current destination	Established = 1 Unestablished and never heard = 2 Unestablished but heard = 3 Not clear = 4
	Accessibility of health resources	The time used to get access to the nearest health institution	Under 15 min = 1 15–30 min = 2 30–60 min = 3 60 min above = 4

*NCMS, New Cooperative Medical Scheme; R&NURMNI, Rural & Non-Working Urban Residents' Basic Medical Insurance; NURBMI, Non-Working Urban Residents' Basic Medical Insurance; UEBMI, Urban Employees' Basic Medical Insurance*.

#### Independent Variable

In previous studies, some scholars described family migration as the family size at the destination and measured it according to the number of family migrants (26). However, these studies only defined migration of couples as family migration, and did not consider whether the children followed (20). Other scholars have taken the nuclear family as the definition (19, 22). This study holds that as long as one family member (including immediate and collateral relatives) moves with the interviewee, this situation could be regard as family migration. On the contrary, if the interviewee moves alone, he/she could be regard as a non-family migration. Therefore, this paper calculates the proportion of local member number of the total family number, and use this proportion which named family migration rate to reflect the degree of family migration.

In addition, most studies consider the nuclear family or husband-wife family and tend to ignore other relatives. This study considered all relatives, but with a slight emphasis. That is, by taking the nuclear family as the benchmark and taking individual migration as the starting point, and focusing on marital relationships, we finally divided family migration into five levels. They are: (a) individual migration, (b) parents or other collateral relatives migrating with the individual, (c) children migrating with the individual, (d) spouse migrating with the individual, and (e) spouse and children migration with the individual, with scores of 1, 2, 3, 4, and 5, respectively. The higher the score, the higher the degree of family migration, which reflects the gradual completeness of the number of nuclear family members and the gradual thickening of blood relationships in the process of family migration.

#### Covariate

The first category is the individual characteristics of the migrant population, including age, gender, subjective health, marriage, education, type of hukou, type of labor contract, and monthly income; the second category is their characteristics of migration, including the migration history, move range, move duration and the amounts of cities' they moved in; the third category is factors of current health resources, including health records and the accessibility of these health resources.

## Results and Discussion

### Descriptive Analysis

#### Current Family Migration Situation

Table 2 show the descriptive statistics of variables. In 2017, among the floating population in China, 31.31% moved to current destination alone, and the remaining 68.69% were migrants accompanied by at least one other person, further verifying that the current population flow in China no longer mainly comprises individuals. In terms of the model of family migration, only 8.5% of the migrant population migrated with their parents or other collateral relatives, 1.44% of the migrant population migrated only with their children, spousal migration accounted for 29.5%, and nuclear family migration accounted for 29.25%, indicating the nuclearization of migrating families. In terms of the family migration rate, the mean is 82.98%, the standard deviation is 0.26, the smallest and largest are relatively 0.1 and 1, means that the proportion of local member of the total family number is relatively high.

**Table 2:** Descriptive statistics of variables (*N* = 71,979).

**Variable**	**Mean**	**SD**	**Min**	**Max**	**Assignment**	**Percent (%)**
Health care	0.41	0.49	0	1	Participated	40.54
					Not participated	59.46
Family migration rate	0.83	0.26	0.10	1		
Age	38.86	9.72	19	88		
Gender	1.44	0.50	1	2	Male	55.76
					Female	44.34
Health	1.16	0.39	1	3	Healthy	85.21
					Basically healthy	13.58
					Unhealthy	1.21
Marriage	1.90	0.70	1	6	Single	20.93
					First married	73.80
					Remarried	1.82
					Divorce	2.09
					Widowed	0.52
					Cohabit	0.84
Education	2.72	1.19	1	5	Primary school or below	12.81
					Junior high school	39.00
					Senior high school	22.90
					Junior college	14.25
					Bachelor degree or above	11.04
Hukou types	1.25	0.43	1	2	Agriculture	74.89
					Non-agriculture	25.11
Labor contract types	2.26	0.72	1	4	Non-fixed-term contract	12.15
					Fixed-term contract	52.76
					Not sign contract	31.36
					Not applicable	3.74
Monthly income	8.17	0.54	0	11.70		
Migration history	10.66	7.31	1	73		
Move range	1.67	0.74	1	3	Across the province	49.75
					Across the city in the province	33.48
					Across the county in the city	16.78
Move duration	74.17	66.95	7	685		
Move cities	1.92	1.67	1	80		
Health records	2.27	1.05	1	4	Established	29.18
					Unestablished and never heard	31.32
					Unestablished but heard	22.77
					Not clear	16.73
Accessibility of health resources	1.19	0.44	1	4	Under 15 min	83.37
					15–30 min	14.92
					30–60 min	1.53
					60 min above	0.18

Table 3 show the heterogeneity of family migration rate among the floating population in China. According to the results, the differences on family migration rate are influenced by age, gender, marriage, hukou type, move range. The male who under 40, well-educated, in marriage status, holding agriculture hukou are more likely to have a higher family migration rate. What is more, people move within province, other than move across provinces, are more willing to relocate with their family member.

**Table 3:** Heterogeneity of Family migration rate by migrants' characteristics (*N* = 71,979).

		**Family migration rate**	
		**Mean %**	**SD**
Age group	<40	85.64^***^	0.249
	≥40	79.16^***^	0.270
Gender	Male	83.25^**^	0.260
	Female	82.64^**^	0.260
Health	Healthy	83.00	0.249
	Unhealthy	82.98	0.260
Marriage	In marriage	79.23^***^	0.273
	Not in marriage	94.61^***^	0.173
Education	Senior high school or below	82.64^***^	0.258
	Senior high school above	83.39^***^	0.265
Hukou types	Agriculture	83.19^***^	0.257
	Non-agriculture	82.35^***^	0.269
Labor contract types	Non-fixed-term contract	83.42	0.260
	Other contract types	82.92	0.260
Move range	Across the province	80.77^***^	0.265
	Move within the province	85.17^***^	0.253
Health records	Established	82.88	0.263
	Unestablished	83.02	0.259
Accessibility of health resources	Under 15 min	82.96	0.260
	15 min above	83.07	0.261

* *p < 0.05*,

** *p < 0.01*,

*** *p < 0.001*.

p means p-value, *means significance level.

#### Health Insurance Participation of Floating Population

Table 4 show the relationships between personal characteristics and medical insurance participation among the floating population in China. First, in terms of individual characteristics, age, gender, health status, marriage status, education, hukou types, and labor contract types will influence the participation of health insurance of floating population. Second, the characteristics of migration such as move range will also influence the participation of health insurance. Third, the health resources characteristics have effect on participation as well, whether establish health records at destination and the distance of the nearest health institution may intervene in decisions.

**Table 4:** Health insurance coverage by migrants' characteristics (*N* = 71,979).

		**Health care**	
		**No**	**Yes**
Age	<40	23,520	18,940
	≥40	19,278	10,239
	*χ^2^* = 710.966, *P* = 0.000		
Gender	Male	24,200	15,938
	Female	18,598	13,243
	*χ^2^* = 26.118, *P* = 0.000		
Health	Healthy	42,167	28,938
	Unhealthy	629	242
	*χ^2^* = 59.52, *P* = 0.000		
Marriage	In marriage	31,841	22,588
	Not in marriage	10,957	6,593
	*χ^2^* = 85.159, *P* = 0.000		
Education	Senior high school or below	37,136	16,643
	Senior high school above	5,662	12,538
	*χ^2^* = 8,100, *P* = 0.000		
Hukou types	Agriculture	35,336	188,568
	Non-agriculture	7,462	10,613
	*χ^2^* = 33,000, *P* = 0.000		
Labor contract types	Non-fixed-term contract	5,608	3,136
	Other contract types	37,190	26,045
	*χ^2^* = 90.296, *P* = 0.000		
Move range	Across the province	20,346	15,461
	Move within the province	22,452	13,720
	*χ^2^* = 205.658, *P* = 0.000		
Health records	Established	11,081	9,920
	Unestablished	31,717	19,261
	*χ^2^* = 551.358, *P* = 0.000		
Accessibility of health resources	Under 15 min	35,473	24,537
	15 min above	7,325	4,644
	*χ^2^* = 18.047, *P* = 0.000		

Table 5 shows the health insurance participation of migrant families in China of 2017. There are 29,181 samples participated in medical insurance at the destination, accounting for 40.54% of the total. Therefore, the enthusiasm of the floating population for participating in insurance at the destination is not very high. This will seriously affect their medical treatment at the destination and make it difficult to address health risks in the migration process. However, only 32.18% of the people who participate in health insurance at the destination are individuals; the vast majority are family migrants. This further indicates that as the floating population gradually completes family migration and achieves family reunification in the destination, they are also seeking opportunities to improve the development capacity and welfare level of their families, including employment, old-age care, medical care, housing, and education, increasing the demand for public services.

**Table 5:** Health insurance coverage of family migrants in China (*N* = 71,979).

	**Health care**			
		**Yes**	**No**	**Total%**
Family migrant	Yes	19,790	29,654	68.69
	No	9,391	13,144	31.31
	Total %	40.54	59.46	100.00

*NCMS, New Cooperative Medical Scheme; R&NURMNI, Rural & Non-Working Urban Residents' Basic Medical Insurance; NURBMI, Non-Working Urban Residents' Basic Medical Insurance; UEBMI, Urban Employees' Basic Medical Insurance*.

### Binary Logistic Regression Analysis

This paper analyzed whether the current family migration trend in China has an impact on the floating population's health insurance participation at the destination. The dependent variable “participation in health insurance at destination” is binary, which could be divide into either participation or non-participation. Thus, we used binary logistic regression model, which constructed as follows:


(1) $$\begin{array}{r} \ln\left(\frac{P}{1-P}\right)=\alpha +\beta_{1}X_{1}+\beta_{2}X_{2}+\cdots +\beta_{i}X_{i} \end{array}$$

The variables were submitted into Stata 16.0 and the binary logistic regression models were established. Model 1 is a benchmark model that only considers personal characteristics control variables; Model 2 considered migration variables and Model 3 considered both migration variables and health resources variables. The regression analysis results show in Table 6.

**Table 6:** Logistic regression results of family migration rate on access to health insurance of floating population in China (*N* = 71,979).

**Variable**	**Model 1**		**Model 2**		**Model 3**	
	**B**	**Exp (B)**	**B**	**Exp (B)**	**B**	**Exp (B)**
Family migration rate	0.435^***^	1.545	0.388^***^	1.475	0.393^***^	1.481
Age	0.004^**^	1.004	−0.013^***^	0.988	−0.013^***^	0.987
**Gender (female)**						
Male	0.139^***^	1.149	0.131^***^	1.140	0.130^***^	1.139
**Health (healthy)**						
Basically healthy	0.039	1.040	0.027	1.027	0.042	1.043
Unhealthy	0.426^***^	1.531	0.363^***^	1.438	0.385^***^	1.470
**Marriage (Single)**						
First married	0.485^***^	1.624	0.430^***^	1.538	0.417^***^	1.517
Remarried	0.663^***^	1.941	0.621^***^	1.860	0.603^***^	1.828
Divorce	0.255^***^	1.290	0.310^***^	1.363	0.301^***^	1.352
Widowed	0.337^**^	1.401	0.425^**^	1.530	0.411^**^	1.509
Cohabit	0.056	1.058	0.035	1.036	0.042	1.043
**Education (primary school or below)**						
Junior high school	0.333^***^	1.396	0.367^***^	1.443	0.357^***^	1.429
Senior high school	0.766^***^	2.151	0.830^***^	2.294	0.811^***^	2.250
Junior college	1.332^***^	3.790	1.414^***^	4.114	1.400^***^	4.054
Bachelor degree or above	1.779^***^	5.921	1.879^***^	6.548	1.869^***^	6.482
**Hukou types (Agriculture)**						
Non-agriculture	0.324^***^	1.382	0.344^***^	1.410	0.336^***^	1.399
**Labor contract types (Non-fixed-term contract)**						
Fixed-term contract	0.992^***^	2.696	1.037^***^	2.821	1.033^***^	2.811
Not sign contract	−1.179^***^	0.308	−1.173^***^	0.310	−1.156^***^	0.315
Not applicable	−0.780^***^	0.459	−0.788^***^	0.455	−0.782^***^	0.458
Monthly income	0.270^***^	1.310	0.195^***^	1.216	0.206^***^	1.228
Migration history			0.026^***^	1.027	0.026^***^	1.027
**Move range (Across the province)**						
Across the city in the province			−0.027^***^	0.973	−0.054^**^	0.947
Across the county in the city			−0.430^***^	0.651	−0.470^***^	0.625
Move duration			0.004^***^	1.004	0.004^***^	1.004
Move cities			−0.061^***^	0.941	−0.062^***^	0.940
**Health records (established)**						
Unestablished and never heard					−0.373^***^	0.688
Unestablished but heard					−0.323^***^	0.724
Not clear					−0.207^***^	0.813
**Accessibility of health resources (under 15 min)**						
15–30 min					−0.087^**^	0.916
30–60 min					−0.089	0.915
60 min above					0.260	1.296
Constant	0.000^***^	0.010	−3.741^***^	0.024	−3.55	0.029
Pesudo *R^2^*	0.229		0.248		0.251	
Log Pseudo Likelihood	−37,438.156		−36,540.233		−36,387.426	

* *p < 0.05*,

** *p < 0.01,*

*** *p < 0.001*.

p means p-value, *means significance level.

### Effects of Variables on Participation in Medical Insurance at the Destination

From Model 1 to Model 3, all of the results reveal that the current family migration trend in China has an impact on the floating population's health insurance participation at the destination. To floating population, their family migration rate is higher, the probability of them to take part in public health care system is higher.

Specially, Model 1 reflects the influence of individual characteristic variables on the floating population's participation in health insurance at the destination. The results show that, in addition to partial health and marriage status factors, other factors have a significant impact on the floating population's medical insurance participation. Then Model 2 considers migration variables based on Model 1, and finds that these migration factors also have great influences on the health care participation. Finally, Model 3 considers both individual, migration and health resources characteristic, it reflects the influence of family migration variables on the participation of the floating population in medical insurance at the destination after controlling for individual, migration and health resources variables.

The statistical results show that family migration rate, the degree of family migration, has a significant positive impact on the insurance participation of the floating population at the destination areas at a significance level of 5%. Exp (B) of family migration rate is 1.481, means that comparing with people with a lower family migration rate, probability of participating in local medical insurance of the people with a higher family migration rate increased by 48.1%.

In terms of individual characteristics, age and labor contract types have a significant negative correlation with participation of the floating population in medical insurance at the destination; gender, health, marriage, education, hukou types, and monthly income have a significant positive correlation. The health care participation rate of people increases 0.987 times as the age increase one unit. Generally speaking, the elderly migrant population tends to be insured. However, the results are inconsistent, which may be due to the new generation of the floating population, comparing to the older generation, have more formal work, and are more eager to settle in cities. They are therefore more willing to participate in health insurance at migration destinations, while the elderly floating population is more mobile and dependent on their hometown. What is more, comparing with floating population who signs a non-fixed or fixed-term labor contract, the people not sign a labor contract has lower probability to participate in public health care system, due to the labor law ask employers to take part in social security system for their signatory employees.

The male migrant population is more inclined to participate in medical insurance, which is 1.139 times the probability of female participation. It may be that the male migrant population faces greater health risks at work and hope to avoid these risks by participating in insurance. Worse health status has a positive effect on health care participation, for the people with terrible body health will take insurance system as a way to keep away from health risks. To marriage status, comparing with single ones, people stay in marriage status, such as first married and remarried, are more possible to take part in health care system. The one who had marriage history also willing to purchase a health care product. Also, the years the floating population has been educated indicates that if people have received good education, they will have higher health literacy and self-protection awareness, so can fully understand medical insurance and are more likely to participate in this security system. Comparing with people who just finished Primary School or Below, the probability of participating in local medical insurance will increased by 42.9, 125, 305, and 548% if people attended Junior high school, Senior high school, Junior college, and even obtained a bachelor degree or above. To those who hold an agriculture type of hukou, they are less possible to take part in health care system at destination because it is complicated for them to trans their health care records from hometown to destination. To economic status, people with relatively high incomes are likely to choose medical insurance to prevent the uncertain risks that the family may encounter. Therefore, the higher the monthly income, the more insurance fee the floating population can afford.

As for migration characteristics, there was a significant correlation between the history, range, duration, cities numbers of migration, and the participation in medical insurance at the destination. The participation rate of floating people whose first move happened many years ago is higher than those whose migration started recently. Move history increases 1 month, the participation rate will increase 2.7%. Maybe the migration history reflects the adapt ability, the longer people move, the more they will adapt to new environment, and then will adjust to current life system. It is the same for the move duration, means the length of the last time move increases 1 month, the participation rate will increase 0.4%. The longer the migration duration, the longer the floating population has been living at the destination, so it is more convenient to participate in and use medical insurance there. In terms of move range, people move across within a province are less likely to participate in health care system at destination. The health care participation rate of people who move across cities in the province and across counties in the city are 0.947 and 0.625 times of people who move across a province, respectively. It may be due to that the longer the distance traveled, the higher the cost of returning to the hometown, so they are more likely to change their institutional welfare status in their place of household registration. However, the more cities people move, the participation rate of floating people is lower. The health care participation rate of people increases 0.94 times as the number of cities they move increase 1 unit.

As for health resources characteristics, members of the floating population who have not established health records are less inclined to participate in medical insurance in the places they migrate to, which is 0.688 times that of people who have established health records, because there is no comprehensive health record management model for the floating population. Also, the accessibility of health resources has a negative effect on health care participation. People will more likely to take part in the public health care system if they can reach the nearest health institution under 15 min, but the health care participation rate will be 0.916 times of the former if people will reach the nearest health institution in 15–30 min.

### Heterogeneity Analysis

Table 7 show the results of heterogeneity analysis. According to the results, in the low-educated group, the effect of family migration rate on health care participation is similar to the effect reflected in total sample, while the effect in high-educated group is proved to be less. To the well-educated floating people, there are much more approaches for them to get access to health resources. In comparison, not well-educated floating people have no more choices than rely on family to collect information at a new environment. When comparing groups by labor contract types, it is obvious that the effect of family migration rate on health care participation is lower in group in which people signs non-fixed-term contract. Maybe the stable and lasting employment status means their health care participation are influenced more by their employers. In terms of income, higher monthly income will help floating people, so the effect of family migration rate on health care participation in the top 50% income group is not as great as in the bottom group.

**Table 7:** Effect of family migration rate on health care participation by education, labor contract types, and income.

	**Model 4**	**Model 5**	**Model 6**	**Model 7**	**Model 8**	**Model 9**
	**Senior high**	**Senior high**	**Non-fixed-term**	**Other contract**	**Top 50% of**	**Bottom 50% of**
	**school or below**	**school above**	**contract**	**types**	**monthly income**	**monthly income**
Family migration rate	0.409^***^	0.280^***^	0.278^**^	0.417^***^	0.224^***^	0.501^***^
	(1.505)	(1.323)	(1.321)	(1.517)	(1.251)	(1.650)
Control variables	Yes	Yes	Yes	Yes	Yes	Yes
*N*	53,779	18,199	8,743	63,235	33,642	38,336
Wald chi^2^	9,237.75	2,585.44	1,474.00	14,252.45	6,215.54	8,394.43
Log pseudo likelihood	−26,755.979	−9,513.8101	−4,661.9372	−31,634.9	−16,595.99	−1,9637.989

* *p < 0.05*,

** *p < 0.01*,

*** *p < 0.001, Exp (B) display in parentheses*.

p means p-value, *means significance level.

Table 8 show the heterogeneity of health care participation among floating families with different family structure. Through calculating the number of family member who under 18 and old who above 59, family structure is divided into 4 types: families without both young and old, families without old but young, families without young but old and families with both young and old. Family migration rate has a significant positive impact on the insurance participation of the floating population at the destination areas at a significance level of 5% in three structure types except for the “families without young but old” structure.

**Table 8:** Effect of family migration rate on health care participation by family structure.

	**Model 10**	**Model 11**	**Model 12**	**Model 13**
	**Families without**	**Families without**	**Families without**	**Families with**
	**both young and old**	**old but young**	**young but old**	**both young and old**
Family migration rate	0.407^***^	0.389^***^	0.388	0.589^**^
	(1.502)	(1.475)	(1.474)	(1.803)
Control variables	Yes	Yes	Yes	Yes
*N*	24,894	44,084	1,136	1,864
Wald chi^2^	5,376.86	9,641.22	212.64	425.85
Log pseudo likelihood	−12,688.507	−22,037.021	−535.183	−958.202

* *p < 0.05*,

** *p < 0.01*,

*** *p < 0.001, Exp (B) display in parentheses*.

p means p-value, *means significance level.

Comparing with Model 10 and 11, the family migration rate in Model 13 has the strongest effect on health care participation, which means that if a family both have old and young family member, the more family members move to the destination, the more likely for them to take part in local health care system. To some extent, the young and the old are so vulnerable that their family have to make a stronger security network, in order to escape from potential disease risks. When the old or young family member come to the new place, the degree of fragile of whole family would increase, so it is a sensible choice to take use of local health care system, especially for families with infants or elderly members.

### Explanatory Mechanism

The logit regression results show that family migration promotes the floating population's participation in medical insurance at the destination. This effect can explained by the mechanisms of family support and social integration.

Firstly, the family support mechanism works because, compared with individual migration, when multiple family members migrate together it is easier to achieve expected income targets, and the burden of medical insurance payments can reduced. Subsequently, the family decides to participate in medical insurance at the destination, which aims to minimize, through diversified family resources, the risk caused by the lack of effective protection at the destination. It is part of the family's risk diversification strategy, which links migration decisions with the maximization of family benefits. In addition, the physical health of the elderly and children may be weaker in general, so the migration of the elderly and children increases the health risks and medical needs of the floating population to a certain extent. Family migrants also facilitate mutual care during treatment, which enhances their willingness to participate in the insurance in the destination.

The second is the social integration mechanism. With a relatively high degree of family migration, especially when all family members migrate, maintaining the welfare system attached to their *hukou* seriously weakens their protection. Family migrants' connection to their hometowns gradually reduces, and they become more closely connected to their destination. They may even wish to settle permanently in cities, which will generate a stronger demand for public services and social welfare there. The floating population no longer intends to return to their hometown, and through family migration, they expand their social network at the destination, enhance their community participation, and facilitate their use of local medical and health services.

**Figure 1:**
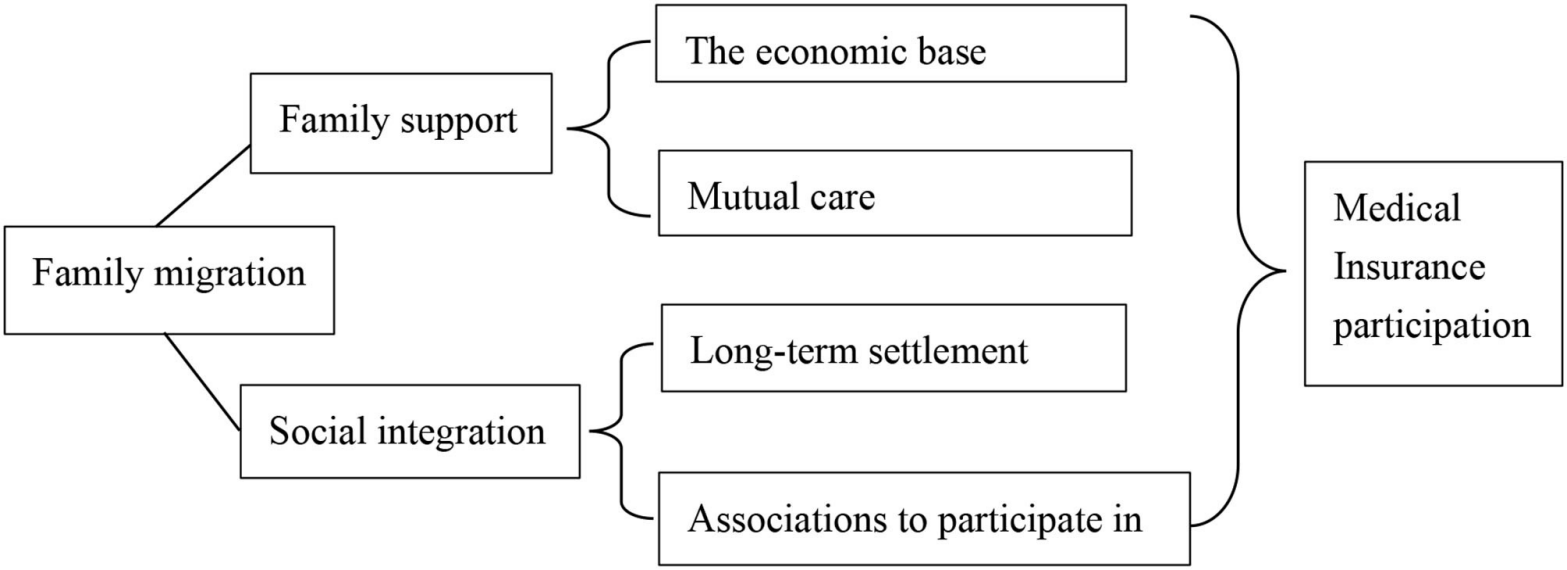
Explanatory mechanisms of family migration's promotion of health insurance participation at the destination.

In summary, the influence mechanism of family migration on the migrant population's participation in medical insurance at the destination concluded below (as shown in Figure 1).

## Conclusions

Population flows in China are shifting from individual migration to partial or complete family migration. This is not only a simple gathering of people in geographic displacement, but also a profound adjustment of family lives and demands of the migrant population. Due to the *hukou* system and regional separation, the floating population in China stay on the margins of the social welfare system and trap in the dilemma of not being able to enjoy basic medical security. In this context, based on the 2017 Migrant Dynamic Survey data, this study used a binary logistic regression method to discuss the family migration rate on health care participation and the influence mechanisms. The final research conclusions are as follows:

First, in 2017, 68.69% of the floating population in China were migrants accompanied by at least one other person, which means that the current population flow in China no longer mainly comprises individuals. In terms of health care participation status, our results shows that the majority of the floating population still chooses to participate in medical insurance in their place of *hukou* registration, and they are in a vulnerable state at the destination, unable to enjoy the same public services as local people.

Second, family migration has a significant positive correlation with the health insurance participation of the floating population at the destination, which explains by family support and social integration mechanisms. The current family migration trend in China has an impact on the floating population's health insurance participation at the destination. Comparing with people with a lower family migration rate, probability of participating in local medical insurance of the people with a higher family migration rate increased by 48.1%. Family migrants have, on the one hand, good family support, including economic foundations and mutual care; and on the other, a relatively high degree of social integration, with a willingness to settle down and enthusiasm to be involved in community life at the destination, which encourages them to participate in medical insurance at the destination.

Third, the participation of the floating population in medical insurance at the destination shows differences according to various characteristics. Age, labor contract types, migration range and cities numbers, health records, and the accessibility of health resources have a significant negative correlation with participation of the floating population in medical insurance at the destination; gender, health, marriage, education, hukou types, monthly income, migration history, and move duration have a significant positive correlation.

Fourth, the heterogeneity of health care participation among different groups within the floating population shows that the effect of family migration rate on health care participation is weaker in group in which people are low-educated and signs non-fixed-term contract or gets bottom 50% monthly income. Also, except for the “families without young but old” structure, the family migration rate of families under other three family structures, especially the “families with both young and old” structure, have stronger effect on health care participation.

Under this trend of family migration, policy-making and institutional arrangements of social welfare face with new challenges. Combined with the results of the empirical analysis and main conclusions, we have following suggestions:

First, we should establish and improve the welfare policies for migrant families to enhance their development ability. As the floating population gradually realizes the migration of complete families, they are no longer isolated. Instead of facing the loss of individual basic rights and interests, they face the loss of the overall interests of their family. Subsequently, the floating population demands basic public services such as employment, education, social security, and public health at the destination. Therefore, relevant social policies should shift from focusing on the floating population to the construction of a public service system centered on family migration. The government will improve the welfare level and expand the welfare coverage of floating population families, in order to respond to their real needs, effectively guarantee their basic welfare, and improve the overall development and security capacity of floating population families.

Second, China should speed up the coordination of medical insurance between the urban and rural areas and expand the coverage of medical insurance. In the development of China's basic medical insurance, regional differences, urban-rural divisions, administrative barriers, and other problems exist, and the portability of medical insurance is inadequate, which makes it difficult for the floating population to participate in and use medical insurance equally. On the one hand, it is necessary to establish the connecting mechanism of basic medical insurance transfer, making full use of information technology; establish multi-level medical security information networks; and simplify the reimbursement procedures in different places. On the other hand, we should strengthen the top-level design, standardize convergence processes and implementation rules, and coordinate procedures among different regions and departments to make convergence policies effective, standardized, and practical.

Finally, we should pay attention to the differences in the characteristics of the floating population and improve their enthusiasm to participate in insurance. Among the floating population, the participation rate of the older generation and female floating population at the destination is low, self-employed workers do not actively participate in insurance, and the Informal employment floating population is often excluded from welfare policy at the destination. Therefore, we should focus on particular groups among the floating population, encourage them to participate in medical insurance, and increase compulsory participation. At the same time, the higher the education level, the more likely the floating population is to participate in insurance. Therefore, education and training of the floating population should strengthen to improve their human capital and health literacy and enhance their participation awareness.

## Data Availability Statement

The original contributions presented in the study are included in the article/supplementary material, further inquiries can be directed to the corresponding author/s.

## Author Contributions

LL contributed to the design and implementation of the research, to the analysis of the results, and to the writing of the manuscript.

## Funding

This research was supported by Nanjing University of Posts and Telecommunications (NYY216005), Jiangsu Provincial Social Science Fund (17SHC006), and Jiangsu Provincial University Philosophy and Social Science Research Fund (2017SJB0079).

## Conflict of Interest

The author declares that the research was conducted in the absence of any commercial or financial relationships that could be construed as a potential conflict of interest.

## Publisher's Note

All claims expressed in this article are solely those of the authors and do not necessarily represent those of their affiliated organizations, or those of the publisher, the editors and the reviewers. Any product that may be evaluated in this article, or claim that may be made by its manufacturer, is not guaranteed or endorsed by the publisher.
